# A cross sectional survey examining the association between therapeutic relationships and service user satisfaction in forensic mental health settings

**DOI:** 10.1186/1756-0500-7-657

**Published:** 2014-09-18

**Authors:** Douglas MacInnes, Helen Courtney, Tracy Flanagan, Daniel Bressington, Dominic Beer

**Affiliations:** Canterbury Christ Church University, Canterbury, UK; Humber Foundation Trust, Hull, UK; Hong Kong Polytechnic University, Kowloon, Hong Kong; Oxleas NHS Foundation Trust, Dartford, UK

**Keywords:** Forensic, Mental health, Regard, Respect, Satisfaction, Therapeutic relationship

## Abstract

**Background:**

This small-scale study examines an often neglected patient group (service users in forensic mental health settings). The research investigates their therapeutic relationship with staff and which therapeutic relationship factors are associated with their level of satisfaction with services.

**Methods:**

A cross sectional survey was undertaken in two medium secure units in the UK with seventy seven participants completing self-report measures examining service user satisfaction with services and their therapeutic relationship with staff. Multiple regression analysis was used to identify the main predictor variables associated with satisfaction with the service provided.

**Results:**

The respondents had a generally positive view of services and also of their therapeutic relationships with staff. However, the therapeutic relationship scores were lower than those recorded in community samples. One predictor variable was significantly associated with service user satisfaction; feeling respected and well regarded.

**Conclusions:**

The therapeutic relationship domain of being respected and well regarded by staff was identified as the most significant factor among the therapeutic relationship domains when examining the association with satisfaction with services. The important role mental health clinicians play in enabling service users to recognize they are being treated respectfully is noted as service users judge the degree of honesty, caring and interest that staff show in them. Staff also need to be available and accessible while having good listening and information giving skills. The importance of having both positive therapeutic relationships and service user satisfaction in forensic settings is also discussed.

## Background

Forensic mental health care is defined as the care of mentally disordered offenders; people who have been in contact with the criminal justice system and who have been transferred to secure hospitals [1]. There are often problems with engagement and staff must meet the therapeutic needs of patients whilst addressing legal, security and public safety issues. The increasing importance of forensic mental health care can be shown by an increase in medium secure unit beds in NHS Trusts in England and Wales from a total of 2,500 in 1997 to 3,723 in July 2007 [1].

The Best Practice Guidelines in Medium Secure Units [2] state the therapeutic alliance between staff and patients is at the centre of high-quality care and treatment in secure settings. This has been most often noted when discussing the importance of the term relational security which has been described as the therapeutic alliance between staff and patients in continuing risk assessment and detailed knowledge of the patient [3]. The Royal College of Psychiatrists [4] have described it as the most important type of security in mental health work as it achieves safety through establishing good rapport and an effective therapeutic alliance between patients and staff. In the Department of Health guide to relational security an appropriate relationship was viewed as being professional, therapeutic, and purposeful with understood limits [5]. This emphasises the need for staff members within a secure setting to be aware of the nature and boundaries of any therapeutic relationship. Developing good therapeutic relationships has the potential for producing clinical and social benefits so it is important to be able to ascertain the ways in which they influence service users’ perceptions of their care and treatment. Dissatisfaction with forensic services is often related to service user’s concerns about therapeutic relationships thus highlighting the importance of this relationship and its association with service satisfaction [6].

### Satisfaction

The National Institute for Mental Health England [7] in their review of forensic mental health services noted that services should seek to build mechanisms and services that involve service users and respond to their views. Measuring service user satisfaction can be viewed as a way of identifying the service user’s perpsective. It is viewed important as successful treatment cannot take place unless there is some satisfaction with the service adding that service user satisfaction provides a way of assessing and monitoring service quality over a period of time [8]. An example of its value can be demonstrated in Brunero and colleagues satisfaction survey of an adult acute in-patient mental health service [9]. They identified a number of areas of need as well as factors that were important predictors of user satisfaction allowing the service to develop interventions to improve service user satisfaction issues raised in the survey.

Until recently, the work looking at service user satisfaction in secure mental health settings has been limited [7]. It was common for results to be recorded indicating that over 90% of respondents were satisfied with their care [10–12] with this attributed to poor research methods thus invalidating the findings [6]. More robust satisfaction surveys have been carried out in adult acute mental health settings concluding that higher amounts of perceived coercion are associated with greater dissatisfaction with formally detained clients less satisfied than those admitted informally [13, 14]. Recent developments in examining satisfaction in secure mental health settings have included the development of more valid measures [15].

### Therapeutic relationships

In mental health care, the therapeutic relationship has been described as the central element of care by which diagnoses are made, care plans negotiated and most interventions delivered [16]. The relationship is the psychological construct held by the individuals participating in the therapeutic relationship. It can last for seconds or decades and have a positive or negative effect. Therefore, the term therapeutic relationship can be viewed as a descriptive expression. What happens when people meet up and engage with each other is a prerequisite for building a relationship and is the cornerstone of mental health care with the culture and atmosphere of a ward determined by what happens in these encounters [17].

From a research perspective, most of the early studies examining therapeutic relationships focused on psychotherapeutic relationships while more recent studies have started to examine the therapeutic relationship in psychiatric settings. However, there are differences in the structure of care in psychiatric settings as opposed to psychotherapy. These include that fact that health professionals often have a statutory role in these settings. Additionally, the clinician often initiates the treatment relationship and the timescale for treatment is usually not fixed. Within psychiatric settings the focus is often more on psychiatric intervention and practical support as opposed to emotional and cognitive processes, and there are often several relationships with different professionals involved in the care process. Thus findings from psychotherapy research are unlikely to be transferrable to these mental health settings.

In research undertaken in mental health settings, the therapeutic relationship has been shown to be influential in predicting positive outcomes in mental health care such as reducing hospitalization, symptomatology and social disability, and increasing engagement with services, medication adherence and global functioning [18–20]. Additionally, the therapeutic relationship, and specifically the degree of helpfulness perceived by the service user, has been viewed as was arguably the most important interpersonal variable in predicting attendance at psychiatric out-patient appointments [21]. A number of commentators have also identified that being non-judgmental, empathetic and respectful positively influences the relationship between staff and service users [22, 23]. However, there is a limited amount of information concerning the therapeutic approaches used in secure mental health settings and whether the therapeutic relationship between service user and staff is an important factor in the service user’s perceptions of their care and treatment [24].

This study examined the following:

- How do service users in a forensic mental health setting view the therapeutic relationship with staff?
- How do service users in a forensic mental health setting view satisfaction with services
- What therapeutic relationship factors are associated with service user satisfaction in secure mental health settings?

## Method

### Design

The study used a cross sectional survey design with participants being asked to complete two self-report questionnaires at one time point.

### Participants

All the service users in two Medium Secure Units (one in London and one in the North of England) were eligible to participate in the study with the only exclusion criterion being that the service user was not able to complete the assessments due to mental capacity. The researchers were guided by the clinical team with the final decision regarding a potential participant’s ability to participate determined by the Consultant Psychiatrist in charge of their care. Potential participants were approached and informed about the project through a variety of methods; the use of posters on the ward, verbal information given at ward meetings, and their key worker talking to them about the study. Each service user was also given written information about the study and the opportunity to discuss any concerns or objections prior to being asked to give informed consent. Anyone expressing an interest in being part of the study was introduced by their key worker to a member of the research team independent of the treating team who obtained informed consent and assisted any service users who requested help to complete the assessments in the clinical area. The participants were paid a nominal amount (£15) for each completed set of assessments as reimbursement for their time and expertise.

### Data collection

Demographic information was requested regarding age, gender, ethnicity and length of inpatient stay. In addition, two assessment measures were completed examining the therapeutic relationship and service user satisfaction.

### Satisfaction

This was measured using the Forensic Satisfaction Scale (FSS) [15]. It is a 60-item measure which asks respondents to rate their satisfaction with a range of services on a five point Likert scale. The scale records levels of satisfaction on seven domains (staff interaction, rehabilitation, communication, milieu, finance, safety, and overall care) as well as giving a total score. The internal reliability of the scale was reported as α = 0.9 with the scale having good concurrent validity with the Verona Service Satisfaction Scale [25]. Mean scores are calculated with higher scores indicating greater levels of satisfaction.

### Therapeutic relationship

The perceived value of the therapeutic relationship was assessed using the revised Helping Alliances Scale (HAS) [19]. This three-item scale assesses: feeling respected and well regarded, beliefs about receiving the right treatment, and feeling understood by clinical staff. The questions are answered via a 100 mm long visual analogue scale with the extreme points being 0 mm = not at all and 100 mm = entirely. Each 10 mm point is marked so it has the appearance of an eleven-point scale. The scores can range from 0 to 100. In addition, to each individual scale score being recorded the average overall score is also sometimes used. The HAS has good reliability and predictive validity. It is reported that the HAS is the most frequently used scale used when measuring the quality of the therapeutic relationship in research evaluating care of people with severe mental illness [26]. The measure had not previously been used to examine therapeutic relationships in forensic mental health settings but was considered a valid and efficient way of measuring the therapeutic relationship between users and staff in secure settings in the absence of any validated measure for use in this setting.

### Analysis

The demographic data were recorded descriptively as were the HAS and FSS scores. Following this, regression analysis was undertaken using SPSS 18 to examine whether the scores of the three HAS domains (feeling respected and regarded, beliefs about receiving the right treatment and feeling understood by clinical staff) were predictive of the FSS total score.

### Ethical considerations

The study was conducted in compliance with the Declaration of Helsinki [27]. Ethical approval was granted by the Faculty of Health and Social Care Research Ethics Committee at Canterbury Christ Church University (Ref: FHSC/024/E) with the study also approved by the relevant Research and Development office for each unit. Informed consent was obtained from all the service-users involved in the study. In order to minimise the risk of participants feeling coerced to take part; the information sheet and consent form clearly stated that participation was entirely voluntary, would have no direct impact upon treatment being received, and that participants had the right to withdraw from the project at any time. Service-users were also informed that the information provided was confidential and the research output would not contain any information that would identify any individual.

## Results

Service users who did not wish to take part were not asked to give a reason for refusal and no distinction was made between those users who discussed their potential participation with the independent researchers and those who did not. Accordingly, the study only details the number of participants and the number of eligible participants. 77 participants completed the two measures out of a possible 139 residing in the units; a response rate of 55.4%. No differences between the two units were recorded in either the HAS or FSS scores. The demographic characteristics of the participants are recorded in (Table 1).

**Table 1:** **Demographic variables**

Category	Number (% of total)
Age 18-25	14 (18.2)
Age 26-35	22 (28.6)
Age 36-45	22 (28.6)
Age 46+	19 (24.7)
Gender Male	65 (84.4)
Gender Female	12 (15.6)
Ethnicity White (a)	45 (59.3)
Ethnicity Black/Other (a)	30 (38.1)
Inpatient stay 0 – 6 months (b)	16 (20.8)
Inpatient stay 7 > 12 months (b)	10 (13.0)
Inpatient stay 1 > 2 years (b)	12 (15.6)
Inpatient stay 2 years plus (b)	34 (44.2)

a = 2 (2.6%) of respondents did not answer this question.

a = 5 (6.5%) of respondents did not answer this question.

The ages of the participants varied with similar numbers of participants from the four identified age ranges. The majority were male 65 (84%) with 12 (16%) female respondents. This is similar to the proportions recorded in medium secure mental health settings in England [28, 29]. In relation to ethnicity, the most frequently recorded was White British while twenty recorded themselves as being of Black British, African or Caribbean origin. The length of in-patient stay varied with the 34 of the participants (44.2%) having been admitted over two years previously and sixteen (20.8%) admitted within the previous six months.

T-tests and ANOVAs were undertaken on the demographic data to ascertain whether there were any differences between demographic factors. To enable a reasonable comparison, the ethnicity of respondents was collapsed to two variables (white and other). No significant differences were found in any of the scores.

The HAS and FSS scores are recorded in (Table 2).

**Table 2:** **HAS and FSS scores**

	HAS respected	HAS treatment	HAS understanding	FSS total score
Mean score (sd)	61.88 (32.24)	63.48 (35.84)	63.29 (32.84)	3.08 (0.45)
Correlation with FSS score (Sig)	0.596	0.520	0.456	n/a

### FSS scores

The total FSS score was 3.08 (sd 0.45). This score was just above the mid-point score of 3 and suggests that there is an overall reasonable level of satisfaction with services being recorded by the users in these two units.

### HAS scores

All three domains on the HAS scored just above 60 suggesting there was general agreement the service users had a positive view of the relationship with clinicians at the units.

There were positive correlations between all three of the HAS domains and the total FSS score indicating that an increase in the therapeutic alliance results in a corresponding increase in service user satisfaction. Although the correlations were all statistically significant (p = < 0.001), the three correlations can all be considered as showing a moderate level of association [30].

### Regression analysis

Multiple regression analysis was performed with the three HAS variables put in as predictor variables and the FSS total score as the dependent variable. The guidance that at least 20 participants were included in the analysis for each predictor variable was adhered to [31]. The ANOVA score F 40.839 df 1 p < 0.001 indicated significant linear relationships between the independent variables and the dependent variable. The results of the regression analysis indicated that these three predictors explained 34.8% of the variance (R^2^ = 0.348) with the only significant predictor the being respected and well regarded variable. All of the predictor variables were positively correlated with the satisfaction score signifying that an increase in a positive perception in the therapeutic relationship was associated with an increase in the satisfaction with the service. The correlations between the three predictor variables ranged from r = 0.653 to r = 0. 722. The standardised beta coefficients ranged from 0.056 to 0.439 while the tolerance values were all well above 0.1 indicating that multicollinearity was not a problem. Using backward regression, the mean being respected score independently predicted 34.7% of the total variance for the FSS total score (Constant = 2.516, R^2^ = 0.347, β = 0.596, t = 6.391, p <0.001). The unstandardized regression co-efficient was 0.08 signifying that for each 10 points scored on the being respected scale, the satisfaction score increases by 0.8.

## Discussion

The results indicated there was a generally positive view of the therapeutic relationship with staff in these secure settings. Due to the lack of studies exploring therapeutic alliances in forensic mental health settings, it is difficult to examine direct comparisons. The HAS scores in this study correspond to a study of community patients [18] which recorded; feeling respected and regarded (66.2 sd 24.7), beliefs about receiving the right treatment (79.5 sd 22.9), and feeling understood by clinical staff (73.5 sd 27.4). Another assessment of users in community services [32] also noted higher HAS scores with the overall average HAS score ranging from 75.09 to 77.27 over a six month period. The indication is the recorded therapeutic relationship scores in this cohort were not as high as those noted by community mental health service users in previous studies. These correspond with the findings of a study of service users in an assertive outreach service [33] which recorded lower scores with an overall HAS score of 63.5 for new patients and 66.6 for established patients and similar to the scores recorded in this study. This suggests that the secure nature of the service and the custodial role that staff members working in forensic mental health settings sometimes perform may influence the nature and perspective of the therapeutic relationship. It also implies the need for clinicians to instigate strategies to promote and maintain the therapeutic relationship is extremely important.

The results also recorded there was overall satisfaction with the services provided which is consistent with other studies. It is near to the findings recorded in previous studies [15, 34] with scores of 3.09 and 3.23 respectively suggesting these satisfaction levels are similar to those in previous studies of medium secure units. In addition, the results supported the view that the therapeutic relationship between service users and staff is a significant element in determining the level of service user satisfaction. The findings indicate that although all three therapeutic relationship domains positively impacted upon service user satisfaction, being respected and well regarded was the most significant factor. This is of major importance to service users in secure settings where they are dependent on staff. It is enhanced in these settings as being respected by staff is one of the essential elements of a caring relationship with service users and plays a large part in the perceptions of the service [17].

### Being respected and regarded

Respect for service user dignity is an important element of identifying good psychiatric care [35]. When service users reported not being respected this led to loneliness and lack of dignity [36, 37] with a lack of respect from staff also viewed as leading to service users disengaging from contact with services [16]. When being respected service users viewed their experiences of the service as positive while, conversely, not being respected resulted in negative opinions being expressed [37].

Service users reported being respected when they were treated as a human being and not as someone inferior. This manifests itself in the ward environment through the perception of being spoken to the same way as other people and being allowed to express their emotions [38]. Service users also reported their worst experience was when they were dismissed as an object rather than someone with basic human rights [39].

The central role of forensic mental health staff in ensuring that service users are treated with respect has been pointed out by many authors noting the important skills required in developing the perception of being respectful of service users. Service users believe respect is linked to a clinicians personal qualities of humanity, equality and being non-judgmental and that they differentiate between staff members perceived as genuinely interested in them and those viewed as uncaring [24, 40]. They are more trusting of, and able to develop therapeutic relationships with, staff members perceived as helpful. Additionally, they view negatively those staff members perceived as uncaring or disinterested and, consequently, disengage with this group of staff. Service users perceive professionals show respect when they are flexible in their approach, when they are supportive despite having conflicting views at times, and by admitting their mistakes when they are wrong [17]. Being honest and trustful was also a major element of showing regard for the service user and this was demonstrated by staff members being true to their word and showing a genuine interest in service users [41].

### Availability of staff

Another important factor identified as a way of showing respect to the service user is the need for staff to be available with the accessibility of staff perceived as a sign that clinicians wish to engage with service users [17]. When staff are not accessible, this is perceived as indicating a lack of interest, which in turn negatively impacts upon the development of positive therapeutic relationships. Staff not having time to engage with service users, and being too distant, are associated with a perceived lack of respect [38]. It has also been observed that interpersonal relationships have to be trustful for a respectful relationship to develop and for this to occur, staff need to spend time with service users [42, 43]. This is supported by previous studies in secure settings where service users reported dissatisfaction about staff members spending limited time with users [40, 44, 45].

### Listening to service user views

The ability to listen to service user views and opinions is also considered important. This is because being listened to is linked to being heard, being treated with respect, and being recognised as a human being [46] while feeling respected is associated with clinicians’ good communication skills including listening, talking and understanding [47]. It has been reported there is a direct relationship between a clinician’s ability to listen and a consequent feeling of respect by service users [48]. It is also suggested that service users want staff to be truly empathetic as this shows they can recognise and anticipate their needs while on a clinical level increased empathy reduces user aggression [49, 50]. Giving correct and relevant information has also been identified as a key clinical skill with service users associating respectful treatment with being given relevant information particularly regarding their treatment or legal rights [38, 42].

### Research in forensic settings

As previously noted, there is only a limited amount of empirical literature looking at therapeutic relationships and user satisfaction in forensic mental health settings. However within these studies, there is some evidence to support the importance of having a positive therapeutic relationship, particularly being respected and well regarded by staff members, and its association with a positive view of the service. A study of women in a medium secure unit, although focusing on the psychotheraputic alliance, found that a positive therapeutic relationship was associated with a positive view of services and also lower levels of security, less behavioural disturbance, and higher levels of motivation and treatment engagement [51]. It was further stated that the therapeutic alliance was a key moderator of the likelihood of inpatient violence and disturbed behaviour. Another study examined the reasons for absconding from forensic mental health units and recorded these were primarily through a sense of boredom or frustration. The conclusions drawn were that a creation of a consistent, transparent and respectful milieu reduced absconding. In addition, these patients were as being a high risk for future violence and that a respectful environment would reduce the risk of violence [52]. Studies undertaken in prison settings have also noted the importance of both therapeutic relationship and satisfaction. A significant relationship between therapeutic relationship and risk factors for violence was found and that staff responsiveness was particularly important for those with at risk of suicide or self-harm. Staff who listened to prisoners needs counteracted the significant frustration, powerlessness and uncertainty that health problems experienced in prison engendered [53, 54]. Although none of these studies directly examined the variables, the strong inference is that a positive therapeutic relationship and high level of service user satisfaction is likely to reduce a range of difficult behaviours and also reduce levels of frustration and powerlessness in the service user group.

The results of this study revealed that being respected and well regarded by staff members was the most important therapeutic relationship factor influencing service user satisfaction in forensic mental health settings. It can be seen that when a service user perceives they are being treated with respect, they feel more positive about themselves, their treatment, and the clinical staff. This results in greater levels of satisfaction with the service. It also allows for a greater degree of discourse surrounding their care and treatment. Conversely, when they do not perceive they are being respected, they have negative views about their experiences and care provided. The availability and accessibility of staff is also important. If staff are often unavailable, they are perceived as being disinterested by service users which in turn leads to a negative therapeutic relationship. For a positive interpersonal relationship to be in place, regular contact is required. The importance of positive therapeutic relationships and service user satisfaction can be denoted by the relationship of these concepts to behavioural disturbance.

### Limitations

There are some potential limitations of the study. The number of participants is relatively small and selected from two units. It is also a cross-sectional study and it is recognised that both therapeutic alliances and satisfaction are both dynamic processes so a study focused on one time point would not be able to capture these developments. In addition, service users and staff may have different perceptions of the therapeutic relationship and satisfaction with services and it may have been helpful to have examined the views of staff as well as service users. Accordingly some caution should be exercised regarding the generalisability of the findings and further work in this area would be useful.

## Conclusions

The findings of this study show that having a positive therapeutic relationship between clinicians and service users is important for service users when judging their satisfaction with the care and treatment received in secure settings. In this cohort, being respected was the one factor found to significantly influence satisfaction. Mental health professionals working in forensic in-patient settings have on-going contact with service users and play an instrumental role in determining service users’ perceptions of the therapeutic relationship. It appears that the user perceiving that staff treating them as equals with individual needs and also accessible and available are the two main areas that impact upon service users’ perceptions as to whether they are being treated with respect or not.

The therapeutic relationship domain of being respected and well regarded by staff was identified as the most significant factor among the therapeutic relationship domains in predicting satisfaction with services. The important role mental health staff play in enabling service users to recognize they are being treated respectfully was noted. Service users judged the degree of honesty caring and interest that staff showed in them. It was also found that being available and accessible were important for the development of a positive therapeutic relationship while listening and information giving skills were important features in showing respect to the service user. In forensic mental health settings creating an environment where staff focus on developing respect and regard for service users may well be a powerful way of reducing behavioural disturbance and increasing the level of therapeutic engagement in these specialist areas.

## Abbreviations

FSS: Forensic satisfaction scale
HAS: Helping alliances scale.
